# Nerve Surgery

**Published:** 1905-10-21

**Authors:** 


### NERVE SURGERY.

**The Surgical Aspect of Trigeminal Neuralgia.** While it is recognised to-day that the most severe form of facial neuralgia can only be cured by surgical measures, it is nevertheless imperative to distinguish other forms of neuralgia which may be alleviated or cured by less severe means. Thus Hutchinson [1] classifies facial neuralgia under four groups. First, that due to abnormal blood conditions—*e.g.* anæmia, gout, or malaria. Second, that due to definite neuritis—*e.g.* tabes dorsalis or herpes frontalis. Third, that caused by some definite local lesion—*e.g.* a carious tooth, inflamed iris, or suppurating antrum. Fourth, epileptiform neuralgia, the cause or pathology of which we know practically nothing. The first three of these groups may be described as neuralgia minor and the last as neuralgia major. As regards the lesser form of neuralgia it is obvious that the all-important point is the recognition of its cause. Carious teeth must be removed, and this, indeed, is generally done in all cases very early, and the good teeth too often are extracted with them, so that such patients are often edentulous before other means are adopted to cure them. Eye strain, glaucoma, iritis, may easily be overlooked, and they may cause severe frontal neuralgia. Syphilitic osteitis and suppuration in any of the accessory nasal sinuses may have severe neuralgia as their most prominent symptom, but they are very amenable to direct treatment if only their existence is diagnosed. But in the most terrible form of all—namely, in epileptiform neuralgia—no adequate exciting cause can be found, and no treatment is permanently curative, except the excision of a part of the root or root ganglion of the fifth nerve. The pain generally begins in and is for some time confined to the distribution of the third division; sometimes the second division is attacked first. The pain may be continuous or intermittent, but is always liable to acute exacerbations, brought on sometimes by cold or movement. The acute attacks last only for a few minutes, but they are of unbearable intensity and leave the constant pain much worse after they subside. Several cases are recorded in which the patients escaped from their sufferings by suicide, and a still larger number have become morpho-maniacs. As regards the pathology of the condition, various observations are so indefinite and contradictory that it may fairly be said that no constant lesion in the nerve or ganglion has been found. Tumours of or involving the Gasserian ganglion cause intense and constant neuralgia of the third nerve, but the pain is not so epileptiform, and there is anæsthesia and some paralysis of the oculomotor nerve. All kinds of medicinal treatment is unavailing in the true neuralgia major. In the early stages of the disease salicylates, bromides, belladonna, gelsemium, and electricity may prove of temporary benefit. Morphia is worse than useless because of the danger of acquiring the morphia habit, which in such cases will assume the most aggravated form. Surgical measures must be adopted if any good is to be done, and provided that the diagnosis is certain, and that all forms of minor neuralgia have been excluded, the sooner they are adopted the better. They include stretching peripheral branches of the nerve—*e.g.* the supra- or infra-orbital nerves; excision of portions of the nerve—*e.g.* the lingual or inferior dental; the removal of the whole of the peripheral branch—*e.g.* the infra-orbital through the antrum or orbit; the removal of the peripheral ganglia—*e.g.* Meckel's with the roots of the peripheral branches; the removal of all the branches of the second and third divisions as near the skull as possible, together with a part of the Gasserian ganglion reached by an enlargement of the foramina of exit of its divisions; and lastly the excision of the whole or part of the ganglion of Gasser by an intra-cranial route. Of these various procedures only three need be considered. Excision of portions of the peripheral branches is so trivial an operation that it may always be tried; and it gives relief for a time, but the pain practically always recurs. If the second and third divisions be reached just outside the skull by Rose's method of turning the zygoma down and the coronoid process up, they may be removed, and the Gasserian ganglion partly picked out through a trephine hole which enlarges the foramen ovale. But this method is very difficult, and there can be no certainty of removing the ganglion, so that as a radical operation it has been superseded by the procedure known as the Hartley-Krause operation. In this a Ω-shaped flap of soft parts and bone is turned down over the temporal fossa. The dura mater covering the temporo-sphenoidal lobe is pushed back, and the Gasserian ganglion discovered in the depth of the wound after tying or pushing aside the middle meningeal artery. Either the whole ganglion with its branches may now be removed, or the outer two-thirds only, leaving the inner third with the ophthalmic division intact. This last detail is important, because if the ophthalmic division is removed the eye will possibly be lost, and the cavernous sinus probably opened; and, moreover, epileptiform neuralgia generally spares this division. But, on the other hand, at the depth of the narrow wound, and embarrassed by constant hæmorrhagic oozing, it is much more feasible to remove the whole ganglion certainly than a definite part of it accurately. And if a patient submits to such a severe measure as this, certainty of radical cure must be the first object aimed at. The integrity of the eye can be safeguarded by sewing together the eyelids, as the first step of the operation, and leaving them thus for ten days. The mortality of the operation when it was first performed was 20 per cent., but lately it has fallen to about 5 per cent. And no case has yet been reported in which the whole ganglion was removed, and in which there has been any recurrence of the pain, even after the lapse of five, eight, or twelve years.

[1] Clin. Jour., April 5 and May 24, 1905.

